# Impact of maintenance therapy using a half dose of the bacillus Calmette–Guérin Tokyo strain on recurrence of intermediate and high-risk nonmuscle invasive bladder cancer: a retrospective single-center study

**DOI:** 10.1186/s12894-020-00766-4

**Published:** 2020-12-09

**Authors:** Dai Koguchi, Kazumasa Matsumoto, Takahiro Hirayama, Shigetaka Moroo, Momoko Kobayashi, Hiroki Katsumata, Masaomi Ikeda, Masatsugu Iwamura

**Affiliations:** Department of Urology, Kitasato University School of Medicine, 1-15-1 Kitasato, Minami-ku, Sagamihara, Kanagawa 252-0374 Japan

**Keywords:** Bacillus Calmette–Guérin, Bladder cancer, Maintenance therapy, Tokyo strain, Nonmuscle invasive bladder cancer

## Abstract

**Background:**

Data are scarce regarding intravesical maintenance therapy (MT) with the low-dose bacillus Calmette–Guérin (BCG) Tokyo strain. We investigated the efficacy and safety of MT with a half dose of the Tokyo strain for patients following transurethral resection of nonmuscle invasive bladder cancer (NMIBC).

**Methods:**

This study retrospectively reviewed clinical data on 78 patients diagnosed with intermediate or high-risk NMIBC followed by either MT (n = 38) or IT alone (n = 40) between January 2012 and March 2018. Statistical analysis was performed to compare recurrence-free survival (RFS) and adverse effects between the two groups. BCG was instilled once weekly for 6 weeks as IT, then once weekly in 2-week for a total of 20 instillations over 3 years.

**Results:**

Kaplan–Meier analyses showed that patients undergoing MT had significantly better RFS than did those undergoing IT alone (hazard ratio (HR):0.32, 95% confidence interval (CI):0.12–0.89, *P* = 0.02). The 3-year RFS was 65.0% in the IT group and 89.5% in the MT group. Multivariate analysis showed that MT was associated with a reduced risk of recurrence (HR: 0.32, 95% CI:0.11–0.93, *P* = 0.03). One MT patient (2.6%) exhibited progression.

**Conclusions:**

The BCG Tokyo strain showed acceptable efficacy and safety in patients undergoing MT; thus, it is a potential treatment for preventing bladder cancer recurrence.

## Background

Reducing recurrence and preventing progression into muscle invasive disease are crucial issues in managing nonmuscle invasive bladder cancer (NMIBC). According to the European Organization of Research and Treatment of Cancer (EORTC) risk tables [1, 2], intermediate and high-risk NMIBC have high recurrence rates ranging from 24%–78% and a high potential risk of 1%–45% for progressing into muscle invasive disease [3]. Intravesical instillation of bacillus Calmette–Guérin (BCG) is currently the most effective immunotherapy for preventing intravesical recurrence and progression after transurethral resection of intermediate-to-high-risk NMIBC and carcinoma in situ (CIS) [4, 5]. Although induction therapy (IT) followed by maintenance therapy (MT) is recommended for optimal efficacy, BCG-related toxicity remains a major clinical problem, resulting in patients discontinuing the therapy [6, 7]. The Southwest Oncology Group study (SWOG8507) evidenced that the optimal protocol for instillation maintenance consisted of 3 weekly cycles with 27 instillations over 3 years. However, only 16% of these patients completed all planned instillations because of severe local and systemic adverse effects (AEs) [8].

Recently, low-dose BCG (LD-BCG) has been shown as a potential option to reduce BCG-associated AEs without affecting the efficacy of full-dose BCG (FD-BCG) for patients following transurethral resection of intermediate-to-high-risk NMIBC [9–11]. For example, a meta-analysis of MT set as 1-yeare period also showed, in comparison with 81 mg the Connaught strain, a significantly better expected overall survival in patients receiving 27 mg with lower probability of AEs [11]. Although such low-dose MT appears promising for treating NMIBC, global BCG shortages due to the sudden cessation of Connaught BCG may limit the MT prevalence. Then, Tokyo strain may be able to overcome the BCG insufficiency because of its’ stable supply, and the Food and Drug Administration is testing its efficacy in a new phase III trial in comparison with that of the Tice strain [13, 14]. However, data are limited regarding the efficacy of MT using the Tokyo strain for LD-BCG, especially with a less intensive regimen than that used in the SWOG [10, 15]. Previously, a phase II study performed by the Kanagawa Urological Research Group (KURG) showed the potential efficacy of MT with FD-BCG based on a 2-week cycle regimen over 3 years after 6 weeks of IT in patients with intermediate and high-risk NMIBC [16]. Therefore, we retrospectively evaluated the efficacy and safety of MT with a half-dose Tokyo strain following the KURG schedule for those who with intermediate and high-risk NMIBC.

## Methods

### Study population

**Fig. 1:**
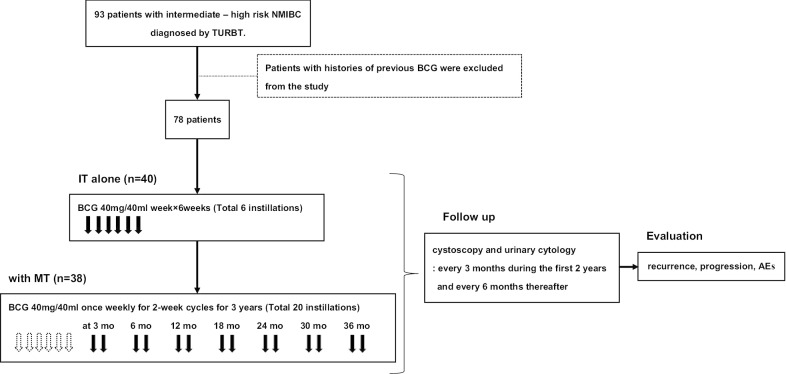
Flow-chart of the study

The study flow chart was illustrated in Fig. 1. The institutional review board at Kitasato University Hospital (B 18-148) approved this study, which was conducted in accordance with the Declaration of Helsinki. This study retrospectively reviewed clinical data on 93 patients diagnosed with intermediate or high-risk NMIBC based on histological examination followed by either MT or IT alone between January 2012 and March 2018. Patients with histories of previous BCG (n = 15) were excluded.

Ta and T1 tumors were completely resected via transurethral resection of bladder tumors (TURBT), and cancers were categorized as one of the following, corresponding to intermediate-to-high-risk in the EORTC risk tables (2): (i) presence of at least two bladder tumors, irrespective of primary or recurrent lesion; (ii) single tumor recurring within 12 months of the previous TURBT for NMIBC; or (iii) any high-grade tumor. In cases of CIS, all primary or recurrent biopsy-proven tumors were included, irrespective of concomitant Ta or T1 tumors, which were completely resected. Exclusion criteria were the presence of lymph nodes or distant metastases, upper tract urothelial or urethral carcinoma, severe infection or any other serious medical complication.

### Treatment

Patients received 40 mg intravesical BCG with the Tokyo strain in 40 ml saline per an instillation during the periods of IT and MT. Treatment method was determined by consultation with the urologist in charge and the patient based on performance status, patient wishes and other factors. BCG solution was instilled into the bladder using a urethral catheter. Patients attempted to retain the solution for 2 h and then voided. The instillation was started between 7 days and 1 month after TURBT. The instillation was performed once weekly for a total of 6 treatments as IT, then once weekly for 2-week cycles at months 3, 6, 12, 18, 24, 30 and 36, for a total of 20 instillations over 3 years, as previously reported by the KURG [16].

### Study methods

Treatment efficacy was assessed based on cystoscopy and urinary cytology findings. Cystoscopy and urinary cytology were performed every 3 months during the first 2 years and every 6 months thereafter. BCG instillation was stopped upon recurrence after TURBT, progression to muscle invasive disease, appearance of CIS, positive urinary cytology results, development of carcinoma in the upper urinary tract or prostatic urethra, or distant metastasis. The completion rate was defined as the number of patients with completed instillations during each period (i.e., months 3, 6, 12, 24 and 36) divided by the total number of eligible patients per group.

The following patient characteristics were collected from the patients’ medical charts: age at time of NMIBC, sex, pathological status (i.e., tumor grade and pT stage), tumor size, number of tumors, recurrence rate, time to recurrence from TURBT, progression rate, mortality, AE types and timing, and reasons for discontinuing MT (categorized as local, systemic AEs and recurrence). AEs were analyzed according to the National Cancer Institute Common Terminology Criteria for Adverse Events, version 4.0. Miction pain, pollakiuria, hematuria, residual urine and urgent urination related to local bladder reactions were categorized as local AEs. Arthralgia, fever of 38.0 °C or higher and rash were categorized as systemic AEs. Tumor grade was assessed per the 1973 World Health Organization (WHO) grading system. Tumor stage was assessed per the 2002 Tumor Node Metastasis classification of malignant tumors.

### Statistical analysis

Chi-square tests were used to compare the two treatment types to identify relationships between the categorical outcome measures, and Fisher exact test was used when sample sizes is < 10. Analysis of variance was used for continuous variables. Recurrence-free survival (RFS) was estimated using the Kaplan–Meier method and compared using the log-rank test. Univariate and multivariate analyses were performed to investigate the efficacy of the low-dose MT. All statistical analyses were performed using Stata, ver. 13 for Windows (Stata, Chicago, IL, USA). All *P-*values were two-sided, and *P* < 0.05 was considered statistically significant.

## Results

Table 1 lists the patients’ characteristics. The study included 61 men (78.2%) and 17 women (21.8%) with a median follow-up period of 36.2 months (interquartile range [IQR]:18.2–55.7). Forty patients (51.3%) received IT alone; 38 (48.7%) received MT. Patient characteristics did not significantly differ between the two groups.

**Table 1:** Comparison of clinical and pathological characteristics between two treatment types with intravesical bacillus Calmette–Guérin

	ALL (n = 78)	Induction (n = 40)	Maintenance (n = 38)	*P* value
Age, years (IQR)	72 (64–78)	76 (67–79)	76 (64–76)	0.36
Sex (%)				
Men Women	61 (78.2)	33 (82.5)	28 (73.7)	0.42
	17 (21.8)	7 (18.5)	10 (26.3)	
pT stage (%)				
pTa/1	60 (76.9)	33 (82.5)	27 (71.1)	0.39
pTis	18 (23.1)	7 (18.5)	11 (8.9)	
Grade (%)				
G1/2 G3	63 (83.3)	35 (87.5)	28 (73.7)	0.16
	15 (16.7)	5 (12.5)	10 (26.3)	
Tumor size (%)				
< 1 cm ≥ 1 cm	45 (57.7)	25 (62.5)	20 (52.7)	0.49
	33 (42.3)	15 (37.5)	18 (37.3)	
Number of tumors (%)				
Single Multiple	14 (17.9)	8 (20.0)	6 (15.8)	0.77
	64 (72.1)	32 (80.0)	32 (84.2)	
*Follow-up, months (IQR)*	36.2 (18.2–55.7)	35.2 (19.3–48.3)	35.9 (17.0–58.4)	0.25

Unless otherwise stated, values are medians with ranges in parentheses or numbers of patients with percentages in parentheses

*IQR* interquartile range

Overall, 19 patients (24.4%) had tumor recurrence, with a significantly lower proportion in MT patients than in IT patients (5 patients [13.2%] vs 14 patients [35.0%], respectively; *P* = 0.025). Of those with recurrence, seven (36.8%) experienced recurrence within 6 months after TURBT, and all had undergone IT alone. None of the MT patients showed recurrence before that period (*P* = 0.046). In subgroup analysis of Ta/1 group (Ta: n = 29, T1: n = 31), there was no significant difference in recurrence between patients with pTa and pT1 (7 patients [24.1%] vs 6 patients [19.4%], respectively; *P* = 0.65). In terms of treatment types, no significant difference in recurrence was found between patients with pTa and pT1 (IT: 6 patients [31.6%] vs 4 patients [28.6%], respectively, *P* = 0.85; MT: 1 patients [10.0%] vs 2 patients [11.7%], respectively, *P* = 0.89).

**Fig. 2:**
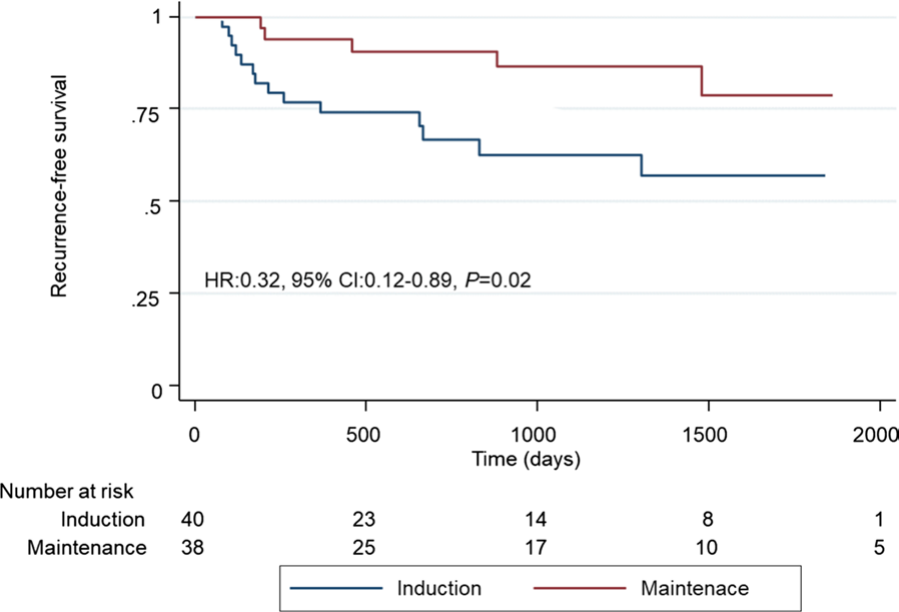
Kaplan–Meier analysis of recurrence-free survival in all patients

**Fig. 3:**
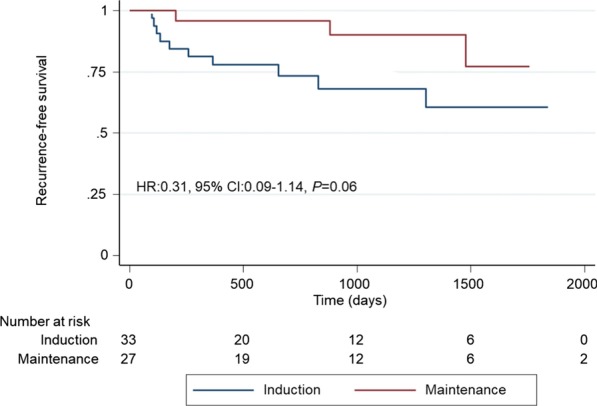
Kaplan–Meier analysis of recurrence-free survival in patients with Ta/1

**Fig. 4:**
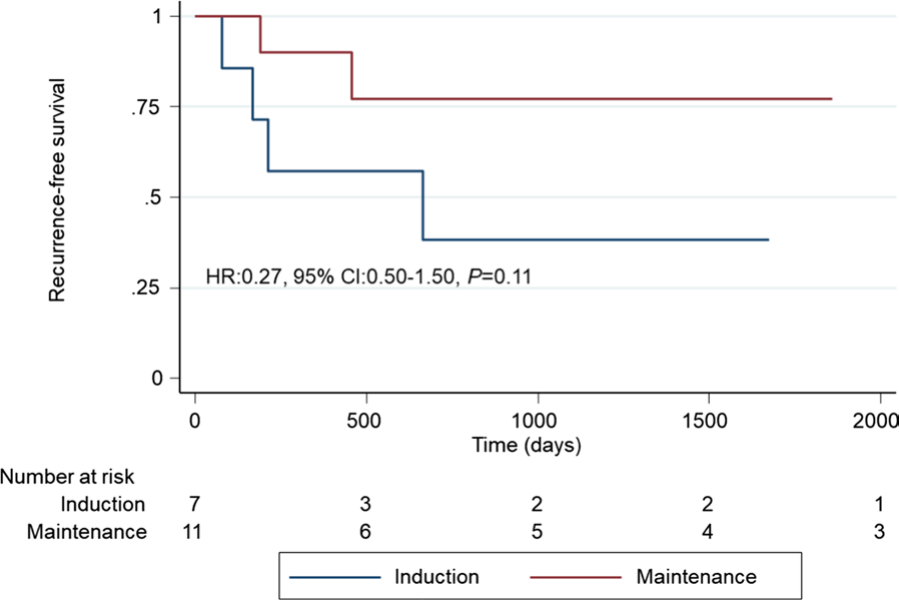
Kaplan–Meier analysis of recurrence-free survival in patients with Tis

Kaplan–Meier analyses showed that MT patients had significantly better RFS than did those who underwent IT alone (hazard ratio (HR) 0.32, 95% confidence interval (CI) 0.12–0.89, *P* = 0.02; Fig. 2). RFS rates in the IT and MT groups were 70.0% and 92.1% at 2 years and 65.0% and 89.5% at 3 years, respectively. In the subgroup analysis, no significant differences occurred between patients with Ta/1 and Tis (HR 0.31, 95% CI 0.09–1.14, *P* = 0.06 and HR 0.27, 95% CI 0.50–1.50, *P* = 0.11, respectively; Fig. 3 and 4). Univariate analysis showed that bladder recurrence was associated with BCG treatment type, and multivariate analysis adjusting for the effects of clinicopathological features showed that MT was an independent risk factor for reduced risk of recurrence (Table 2).

**Table 2:** Univariate and multivariate analyses for recurrence-free survival

Variable	Category	Univariate analysis			Multivariate analysis		
		HR	95% CI	*P* value	HR	95% CI	*P* value
BCG treatment	Maintenance	0.31	0.11–0.88	0.03	0.32	0.11–0.93	0.03
	Induction	Ref			Ref		
Age		0.82	0.31–2.17	0.70	0.58	0.20–1.64	0.30
Sex	Women	0.73	0.43–1.26	0.28	1.35	0.30–6.13	0.70
	Men	Ref			Ref		
T stage	Ta/1	1.51	082–2.79	0.20	0.78	0.20–2.95	0.71
	Tis	Ref			Ref		
Grade	G1/2	1.39	0.70–2.77	0.36	1.92	0.47–7.80	0.36
	G3	Ref			Ref		
Size	< 1 cm	0.93	0.55–1.57	0.79	0.38	0.13–1.39	0.84
	≥ 1 cm	Ref			Ref		
Number of tumors	Single	0.74	0.37–1.48	0.40	0.48	0.13–1.81	0.28
	Multiple	Ref			Ref		

*HR* hazard ratio, *CI* confidence interval, *BCG* bacillus Calmette–Guérin

One patient (2.5%) in each group experienced tumor progression, with locally advanced progression in the IT patient diagnosed with pT1 and lymph node metastasis in the MT patient with pTis. Both patients had subsequent intravenous chemotherapy for the progression. Six MT patients (7.7%) died: one with tumor progression died of bladder cancer, and the remaining five died of respiratory failure, gallbladder cancer, cervical cancer, esophageal cancer and malignant lymphoma.

Table 3 lists the AEs. A significantly greater percentage of patients had AEs in the MT group than in the IT group (68.4% vs. 32.5%, *P* = 0.003). One patient with IT experienced two systemic AEs: arthralgia and fever. No AEs were classified as > grade 3 in either group. The percentage of patients with AEs during the induction period did not significantly differ between the two groups (MT, 12 patients, 31.6%; IT, 12 patients, 32.5%; *P* = 0.93). In the MT patients, 88.5% (23/26) experienced AEs until the first 6 months of the maintenance period. Common symptoms were miction pain (36.8%) and fever (23.7%).

**Table 3:** Comparison of adverse effects between two treatments types with intravesical bacillus Calmette–Guérin

	ALL (n = 78)	Induction (n = 40)	Maintenance (n = 38)	*P* value
*Adverse effects (%)*				
Grade ≤ 2/Grade ≥ 3	39 (50.0)/0	13 (32.5)/0	26 (68.4)/0	0.003
Local	30 (38.6)	9 (22.5)	21 (57.9)	0.003
Systemic	16 (20.5)	7 (17.5)	9 (23.7)	0.50
Both	7 (9.0)	3 (7.5)	4 (10.5)	0.64
*Local symptoms (%)*				
Miction pain	18 (23.1)	4 (10.0)	14 (36.8)	< 0.001
Pollakiuria	8 (10.3)	3 (7.5)	5 (13.2)	0.41
Hematuria	6 (7.7)	1 (2.5)	5 (13.2)	0.10
Residual urine	2 (2.6)	0	2 (5.3)	0.23
Urgent urination	1 (1.3)	1 (2.5)	0	0.33
*Systemic symptoms (%)*				
Arthralgia	1 (1.3)	1 (2.5)	0	0.33
Fever	15 (19.2)	6 (15.0)	9 (23.7)	0.40
Rash	1 (1.3)	1 (2.5)	0	0.33

Unless otherwise stated, values are numbers of patients with percentages in parentheses

Maintenance BCG was instilled in 35 patients (92.1%) at month 3, in 27 patients (71.1%) at month 6, in 22 patients (57.8%) at month 12, in 15 patients (36.8%) at month 24, and in 7 patients (18.4%) at month 36. Twenty-four patients stopped MT due to toxicity (12 patients, 31.6%; 10 local AEs and 2 systemic AEs), recurrence (3 patients, 7.9%) and others (9 patients, 23.7%). The remaining seven patients (18.4%) remained on MT at the time of this analysis.

## Discussion

In this study, MT was performed with a half dose of the Tokyo strain and was continued in 2-week cycles. Data regarding this regimen are extremely limited, even in the era of a BCG shortage [3, 9–11]. We retrospectively investigated the efficacy and safety of MT with LD-BCG for patients following TURBT for intermediate-to-high-risk NMIBC. Patients undergoing MT showed significantly better RFS compared with that of IT alone, and multivariate analysis showed that only MT was associated with a reduced risk of recurrence. Although the overall toxicity with MT was 68.4%, including local and systemic AEs, no AEs were classified as ≥ grade 3, and the discontinuation rate of MT due to toxicity was only 31.6%.

Many studies have investigated the feasibility of LD-BCG [3, 9]; however, data regarding LD-BCG using the Tokyo strain for patients following TURBT for NMIBC are scarce. With IT, previous cohort studies attained equipotent efficacy at a half dose of the Tokyo strain compared with its standard dose [17, 18]. Irie et al. reported no significant difference in RFS [17], and a historical cohort study including 156 patients showed no significant differences in RFS or progression-free survival [18]. Only one study analyzed the efficacy of 40 mg of the Tokyo strain as MT [15]. Akaza et al. performed an RCT in which LD-BCG was added to the IT and injected once monthly for a year (totaling 12 instillations), with a 3-year RFS of 77.8%. In addition to these favorable results of the half dose of the Tokyo strain, both in vitro studies and clinical trials have shown that the Tokyo strain could be an alternative to the Connaught strain thus, the Tokyo strain requires further study, as it may contribute to the stable supply of BCG [19–21].

The widely accepted maintenance protocol for the last 2 decades has been based on SWOG8507. This protocol consists of FD-BCG with 3-week cycles for 3 years, totaling 27 instillations. However, recent evidence suggests that the SWOG regimen may include unnecessary BCG instillations [3, 9]. A meta-analysis recently indicated a nonlinear association between the dose per instillation and clinical efficacy [9]. A large phase III RCT (EORTC30962) found that the 5-year RFS rates were 54.5%, 58.8%, 62.6%, and 64.2% for 1/3-dose/1 year, full-dose/1 year, 1/3-dose/3 years, and full-dose/3 years, respectively (*P* = 0.038) [22]. Compared with a large RCT based on SWOG8507, the present study with 2-week cycles for 3 years with 20 instillations in total showed acceptable RFS. The 2-year RFS was 92.1%, and the 3-year RFS was 89.1% in the present study, while the 2-year RFS was 82% in SWOG8507, and the 3-year RFS was 65.0% in EORTC30911 [8, 23]. The KURG study supported the potential efficacy of the 2-week cycle regimen. This prospective phase II study of MT with FD-BCG followed the same instillation schedule as ours, showing a 3-year RFS of about 80% [16]. To date, although controversies surround the efficacy of MT with LD-BCG, the American Urological Association suggested reducing the BCG dose from an economical point of view [24–26]. Considering the recurrence, progression rate, and toxicity, the present study implied a favorable cost-effectiveness of MT using a half dose compared with the FD-BCG. Further large prospective studies are needed to find an optimal reduced-intensity regimen in terms of dose and frequency using various BCG strains.

Previous studies have evidenced positive efficacy with the 2-week cycle [27–29]. A prospective study found that high urinary leukocytes predicted MT efficacy in patients with NMIBC and a level of urinary leukocytosis and Th1 peaked at around the second instillation. [27, 28]. A recent analysis revealed increased blood platelets after 2–4 BCG instillations in patients with NMIBC. These instillations may introduce leukocyte infiltration into the stromal tissue and modulate the effector functions of neutrophils and macrophages [29]. Although the optimal MT schedule is unclear [3, 9, 11], these patterns of immunological responses to intravesical BCG instillations reinforced the clinical outcomes of the present study based on the 2-week cycle.

LD-BCG has been used in efforts to reduce AEs and secondarily improve MT completion rates [9, 10, 30]. Nevertheless, compared with previous studies using FD-BCG for the same 3-year period, the present study with the half-dose Tokyo strain showed a similar toxicity rate with a lower completion rate. Local and systemic AE rates were 57.9% and 23.7% in the present study, 63.3% and 33.6% in the EORTC30962, and 67.5% and 22.5% in a recent large retrospective study in Japan, respectively [31, 32]. The completion rate was 18.4% in the present study and 35% in both KURG and EORTC3092 [16, 31]. However, in the present study, no patients had AEs above grade 3, compared with about 10% in the KURG study [16]. Furthermore, the main reasons for discontinuation were unrelated to physical AEs. These reasons included drop-out and patients’ preferences, which accounted for 23.7%. Hence, physical AEs may not always accurately reflect the MT discontinuation [33]; this is supported by accumulating evidence showing that most AEs during MT occur within the first year, then decrease gradually thereafter in inverse proportion to the number of patients discontinuing MT [31, 32]. Therefore, further analyses of toxicity, including emotional aspects such as patients’ quality of life, should be analyzed to increase the completion rate.

This study had some limitations. First, the study was retrospective, and the association between previously collected data and clinical outcomes in the current study may result from unmeasured or residual confounding by other factors. Second, the WHO 1973 classification might require a careful understanding of the diagnosis of particularly Grade 2 diseases. Third, statistical analyses of clinical outcomes other than RFS were insufficient; however, only one patient per group progressed to bladder carcinoma, and only one MT patient died of bladder carcinoma. A longer follow-up may enable performing appropriate statistical analyses of such events with larger numbers. Forth, seven patients remained on MT at the time of this analysis; four of seven patients had completed over 2 years of follow-up, and two of the remaining three patients completed over 1 year of follow-up. Thus, we believe that the follow-up periods were sufficient to evaluate the clinical outcomes.

## Conclusions

The present study using the half-dose Tokyo strain and 2-week cycles showed acceptable clinical outcomes with no AEs classified above grade 3 in the MT group. Furthermore, multivariate analysis showed that MT was an independent reduced risk factor of recurrence. Therefore, the present reduced-intensity regimen relative to that of the SWOG should be strongly considered during a BCG shortage and should be validated in further large prospective studies that also evaluate patients’ emotional aspects for better managing maintenance periods.

## Data Availability

The datasets used and/or analysed during the current study are available from the corresponding author on reasonable request.

## Abbreviations

AEs: Adverse effects
BCG: Bacillus Calmette–Guérin
CIS: Carcinoma in situ
CUETO: Club Urológico Español de Tratamiento Oncológico
CI: Confidence interval
EORTC: European Organization of Research and Treatment of Cancer
FD-BCG: Full-dose BCG
HR: Hazard ratio
IT: Induction therapy
KURG: Kanagawa Urological Research Group
LD-BCG: Low-dose BCG
NMIBC: Nonmuscle invasive bladder cancer
MT: Maintenance therapy
RCTs: Randomized control trials
RFS: Recurrence-free survival
SWOG: Southwest Oncology Group
TURBT: Transurethral resection of bladder tumors
WHO: World Health Organization
